# *In Vivo* Interrelationship between Insulin Resistance and Interferon Gamma Production: Protective and Therapeutic Effect of Berberine

**DOI:** 10.1155/2016/2039897

**Published:** 2016-08-24

**Authors:** Mohammad Ahmad Mahmoud, Doaa Ahmad Ghareeb, Heba Abdelghany Sahyoun, Ashraf Abdelhamed Elshehawy, Mohammad Mohammad Elsayed

**Affiliations:** ^1^Chemistry Department, Faculty of Science, Kafrelsheikh University, Kafr El-Sheikh, Egypt; ^2^Biochemistry Department, Faculty of Science, Alexandria University, Alexandria, Egypt

## Abstract

This research was conducted to investigate if there is a relation between insulin resistance incidence and inhibition of interferon gamma production or not. Firstly, insulin resistance was induced by high fat diet (HFD) intake for 6 weeks. Secondly, berberine was used as protective/curative compound for insulin resistance. Results revealed that feeding rats HFD for 6 weeks developed features of insulin resistance (IR) syndrome. These features presented in increased body weight, hyperglycemia, hyperinsulinemia, hypercholesterolemia (with increased LDL-cholesterol and decreased HDL-cholesterol), and hypertriglyceridemia. Level of antioxidant enzymes in HFD group was higher than in normal one. Also there was an increasing in level of proinflammatory cytokines as interleukin- (IL-) 6 and IL-12 in HFD group. Feeding rats HFD for 6 weeks also decreased level of interferon gamma (IFN-*γ*). The decreased level of IFN-*γ* has been shown to predict infection with infectious diseases especially viral infection. Treatment and protection with berberine 50 mg/kg/day for 2 weeks were found to be effective against the features of insulin resistance syndrome, improved levels of insulin resistance parameters, lipid profile, antioxidant enzymes, proinflammatory cytokines, and IFN-*γ*.

## 1. Introduction

The insulin resistance (IR) or metabolic syndrome (MS) is characterized by the clustering of metabolic abnormalities including central obesity, atherogenic dyslipidemia, hypertension, and systemic insulin resistance that leads to an increased risk for type 2 diabetes (DM2) and cardiovascular disease (CVD) [1–3].

IR is a pathological condition in which cells fail to respond to the normal actions of the hormone insulin. IR results from defects at the level of ligand-receptor-response pathway which occur either at the receptor level or in insulin receptor substrate (IRS) molecules by decreasing signaling proteins levels or modulating their activity by phosphorylation.

Due to the change in insulin signaling pathway which occur during IR, several pathophysiological changes took place such as glucose intolerance, obesity, dyslipidemia, and hypertension. Furthermore, several inflammatory cytokines and lipid metabolites like free fatty acids which increased with IR and induced type 2 diabetes mellitus [4].

Interferons (IFNs) are glycoproteins made and released by host cells in response to the presence of pathogens, such as viruses, bacteria, parasites, or tumor cells [5]. In contrast to antibodies, interferons are not virus specific but host specific. Thus, viral infections of human cells are inhibited only by human interferon [6].

Obesity is a major risk factor for cardiovascular disease, certain types of cancer, and type 2 diabetes. The development of type 2 diabetes begins with the onset of insulin resistance, which is correlated with the expansion of visceral fat mass [7]. Obesity is associated with increased release of the proinflammatory cytokines tumor necrosis factor- (TNF-) *α* and interleukin- (IL-) 6 from adipose tissue, which leads to a state of chronic inflammation [8, 9].

IL-6 plays an important role in the differentiation of several cell types. IL-6 upregulates suppressor of cytokine signaling 1 (SOCS1) expression in activated cluster of differentiation (CD) 41 T cells, thereby interfering with signal transducer and activator of transcription 1 (STAT1) phosphorylation induced by interferon *γ* (IFN-*γ*). Inhibition of IFN-*γ* receptor-mediated signals by IL-6 prevents autoregulation of IFN-*γ* gene expression during CD41 T cell activation, thereby preventing T-helper- (Th-) 1 differentiation. Thus, IL-6 promotes CD41 Th2 differentiation and inhibits Th1 differentiation by two independent molecular mechanisms [10]. Therefore, IR alters the immune system response because it shifts T-helper differentiation toward Th-2 and prevented the Th1 differentiation which is responsible for the cytotoxic T-lymphocytes response. The action of IR in immune system is similar to hepatitis C virus (HCV) effect [10].

Nature has been a source of medicinal agents since the beginning of time. The World Health Organization (WHO) estimates that herbal medicine is still the most common source for primary health care of about 75–80% of the world's population, mainly in the developing countries, because of better cultural acceptability, better compatibility with the human body, and fewer side effects [11].

*Berberis vulgaris* L. is considered as one of the well-known medicinal plants with traditional herbal medical history and used by many civilizations as a curative herbal remedy in homeopathic systems of medicine [12]. The identification of certain alkaloids and phenolic compounds in barberry somehow provides an alternative method for medicine and remedies. Those therapeutic compounds could lead to the development of new drugs derived from that plant, which is believed to be safer and more effective [13].

On one hand, berberine (BRB, the most abundant alkaloid found in* Berberis vulgaris*) was shown to decrease blood glucose, enhance insulin sensitivity, and reduce weight gain in both dietary and genetic rodent models of type 2 diabetes. In high fat diet induced obese rats, berberine decreased fasting blood glucose (FBG), postprandial blood glucose (PBG), fasting insulin, homeostasis model of assessment-insulin resistance (HOMA-IR), and body weight [14–16]. Berberine activates the adenosine monophosphate activated protein kinase (AMPK) which reduces IR through enhancement of adipocytes' glucose uptake. Berberine also increases the expression of hepatocytes insulin receptor and improves cellular glucose consumption [17]. Furthermore, BRB increases glucose transporter-4 (GLUT4) translocation in adipocytes [14]. Moreover, berberine activates the extracellular signal regulated kinase pathway which in turn increases the LDL receptor (LDLR) expression at the posttranscriptional level [17]. Also, berberine reduces hepatocytes lipid synthesis through AMPK activation and decreases peroxisome proliferator-activated receptors- (PPAR-) *α* mRNA and protein expression, which emphasizes the berberine-hypotriglyceridemic effect [17].

On the other hand, berberine has immune-modulation effect where it has been reported that treatment of macrophages and dendritic cells (DCs) with berberine significantly induced the expression and production of interleukin-12 (IL-12), which consequently increased the interferon gamma (IFN-*γ*) production and decreased the IL-4 level in antigen-primed CD4C T cells [18]. Therefore, berberine has a stimulatory effect on T-helper lymphocytes subset 1 (Th1) cytokine synthesis in CD4C T cells and an inhibitory effect on Th2 [18]. In case of viral infections as HCV, immune system cannot eradicate the infection as HCV proteins subverts cellular response of the host; it also causes imbalance in TH1/TH2 ratio and targets the body in TH2 way and antibody production which cannot clear the viral particles due to its heterogenecity.

Therefore, the goal of our work was to prove the relation between insulin resistance incidence and the inhibition of interferon gamma production and then assess the effect of berberine as protective and curative compound against IR on interferon gamma production in order to develop a new therapeutic regimen for infection diseases as HCV.

## 2. Materials and Methods

Trichloroacetic acid (TCA), 5, 5′-dithio-bis-2-nitrobenzoic acid (DTNB), thiobarbituric acid (TBA), reduced glutathione (GSH), and sulphanilic acid were purchased from Sigma-Aldrich (USA). Kits of cholesterol, HDL-cholesterol, and triglyceride were purchased from Biosystems (Spain), glucose was purchased from Spinreact (Spain), ELISA kit of insulin and IFN-*γ* were purchased from DRG (USA) and Komabiotech (Korea), respectively, and IL-6 and IL-12 were purchased from Sunredbio (Shanghai). All other chemicals and reagents were of analytical grade.

### 2.1. Animals

Female rats were purchased from experimental animal house, Faculty of Medicine, Alexandria University. The rats aged about 6–8 weeks old and weighted (130–140 g) were used in this study. The animals were housed (six rats/cage) and they were allowed free access to pelleted food and tap water for one week before treatment. All animals were maintained at approximately 23°C–25°C with a 12 h light/dark cycle. Experiments were performed following international ethical standards and according to the Guide for the Care and Use of Laboratory Animals of the National Institutes of Health [19].

### 2.2. Diet

The composition and preparation of HFD were described in Table 1. HFD contained 58% fat, 25% protein, and 17% carbohydrate, as a percentage of total kcal. HFD was freshly prepared on a weekly basis and stored at 4°C, while normal basal diet (NPD) contained 11% fat, 16% protein, and 15% carbohydrate (Table 1) [20].

### 2.3. Animal Treatment

The experimental animals were divided into 4 groups each consisting of 6 rats, in Group 1 (control group), healthy rats received normal diet and tap water for evaluation of the basal level of all the experimental parameters, in Group 2 (untreated HFD), rats received HFD for 6 weeks, in Group 3 (treated HFD with BBR), rats firstly received high fat diet for 6 weeks and then received oral administration of berberine daily at a dose of 50 mg/kg/day for 2 weeks, and in Group 4 (protected HFD with BBR), rats firstly received basal diet with oral administration of berberine daily at a dose of 50 mg/kg/day for 2 weeks and then received HFD for 6 weeks.

All animals were fasted for 12 hours before scarification but after 6-hour starvation period blood sample was collected for blood glucose level determination. After complete anesthesia, the abdominal cavity was rapidly opened following the median line of the abdomen. Blood was collected in plain tubes to get sera (for biochemical analysis) and ethylenediaminetetraacetic acid (EDTA) tubes for whole blood (for complete blood count (CBC) analysis) from all groups. The sera tubes were kept at room temperature for 15 min for blood clotting and centrifuged at 3000 r.p.m. for 10 minutes; then the obtained sera were kept at −20°C until analyzed.

The liver from each rat was removed, washed in cold saline, and cleaned. The tissues were weighed, cut into small anatomical pieces, and homogenized to obtain 10% homogenate (1 gm of liver pieces was homogenized in 9 mL cold phosphate buffer, pH 7.4, 0.1 M, and centrifuged for 15 min at 4000 rpm, 4°C, to remove the cell debris).

The standardized methods for determination of serum glucose level [21], blood insulin level [22], HOMA-IR index [23], serum cholesterol level [24], serum triglyceride level [25], serum HDL-cholesterol level [26], serum LDL-cholesterol level [27], liver thiobarbituric acid reactive substances (TBARS) [28], liver glutathione-S-transferase (GST) activity [29], and liver reduced glutathione (GSH) content [30] were used. Moreover, serum Nitric Oxide (NO) level, serum IL-6 level, serum IL-2 level, and serum INF-*γ* level were carried out using commercial kit according to manufactures' instructions.

## 3. Statistical Analysis

Data were analyzed by one-way analysis of variance (ANOVA) using Primer of Biostatistics (Version 5) software program. Significance of means ± SD was detected groups by the multiple comparisons Student-Newman-Keuls test at *p* < 0.05.

## 4. Results

Table 2 shows that feeding rats HFD for 6 weeks significantly (*p* < 0.05) increased body weight, blood glucose, insulin, and HOMA-IR levels by 19.67, 36.31, 98.18, and 175.69% while decreased HOMA-*β* level by 32.77%. Treatment with berberine normalized body weight, glucose, insulin, HOMA-IR, and HOMA-*β* levels, at *p* < 0.05, while protection with BRB normalized all parameters except body weight which is still high like untreated HFD group weight at *p* < 0.05.

There was a marked significant increase in lipid profile of HFD group than that of control one where the cholesterol, TG, and LDL levels were significantly increased by 101.9, 141.15, and 323.84% over control level, while HDL level was decreased by 25.53% than that of control group, at *p* < 0.05. Both treatment and protection with BRB significantly reduced level of serum cholesterol, triglycerides, and LDL-cholesterol and elevated HDL-cholesterol when compared with HFD group by the same level but failed to normalize them, at *p* < 0.05 as shown in Table 3.

Table 4 reveals that there was a significant (*p* < 0.05) elevation in the level of IL-6 in untreated HFD group when compared to control group. Both treatment and protection with BRB improved the level of IL-6 when compared to HFD but did not normalize it. The level of IL-6 in protected group was higher than that of treated group, at *p* < 0.05.

HFD intake decreased the level of IL-12 when compared to control group, at *p* < 0.05. Both treatment and protection with BRB normalized the level of IL-12, at the same level.

Also, there was a significant (*p* < 0.05) decrease in the level of IFN-*γ* in untreated HFD group when compared to control group. Both treatment and protection with BRB improved the level of IFN-*γ* when compared with HFD but did not normalize it.

While there was no significant difference in the count of WBCs among all tested groups, HFD intake decreased lymphocytes number comparing with control one, at *p* < 0.05. Both treatment and protection increased lymphocytes number than those of HFD group by the same level but failed to normalize it.

Table 5 reveals that feeding rats HFD elevated TBARS, GSH, GST, and NO levels when compared to control group. Both treatment and protection with BRB normalized all these parameters, at *p* < 0.05.

## 5. Discussion

It is reported that IR is associated with increased release of the proinflammatory cytokines TNF-*α* and IL-6 from adipose tissue, which leads to a state of chronic inflammation, and also stimulated production of a family of proteins known as suppressors of cytokine signaling (SOCS) via IL-6 that participate in a negative feedback loop in cytokine signaling [31–33]. This study was aimed to find out the relation between IR and INF-gamma production and study the modulation effect of berberine.

Our study demonstrated that feeding rats a HFD for 6 weeks resulted in development of features of insulin resistance syndrome. These features presented in increased body weight, hyperglycemia, hyperinsulinemia, hypercholesterolemia (increased LDL-cholesterol and decreased HDL-cholesterol), and hypertriglyceridemia. Also, feeding rats HFD for 6 weeks and induction of IR decreased level of IFN-*γ*. These findings emphasized the direct correlation between IFN-*γ* and IR incidence.

With the induction of IR, there is an elevation in the level of proinflammatory cytokines as IL-6 and IL-12 when compared to healthy rats. These results are supported by other studies that showed that adipose tissue has been shown to produce 10–35% of IL-6 in a resting individual, and this production increases with increased adiposity [34]. This suggests that adipose tissue is a source of the increased circulating IL-6 observed in obesity. Also, Senn et al. [35] showed that high level of IL-6 in obese individual induces insulin resistance.

Also, the study of Diehl et al. [10] reported that there is slight reduction in level of IL-12 in cells affected by IL-6 but this reduction was not reproducible.

Interferon *γ* is a strong activator of inflammatory responses and cellular immunity and is a major activator of macrophages [36]. IFN-*γ* is produced predominantly by NK, Th1, and CD8^+^ cells and binds to a heterodimeric cell surface receptor that is ubiquitously expressed. Ligation of the IFN-*γ* R results in activation of the receptor-associated tyrosine kinases Janus kinase 1 (Jak1) and Jak2, leading to the tyrosine phosphorylation and activation of the transcription factor STAT1 [36, 37].

In the present study the induction of IR increased the level of IL-6 and reduced the level of IFN-*γ* and the number of lymphocytes compared to normal one but there is no effect on total WBCs count when compared to healthy rats. These findings seem to be in concordance with the study of Diehl et al. [10] that showed the effect of IL-6 high level on IFN-*γ* production.

IR, a hallmark of type 2 diabetes, is associated with oxidative stress. Although there is substantial evidence that hyperglycemia results in the generation of reactive oxygen species (ROS) and increased oxidant stress in the late complications of diabetes [38], the role of ROS in the development of insulin resistance [39, 40], especially at the whole-body level, remains virtually unknown. Levels of antioxidant enzymes are greater in obese insulin resistant group than in normal one; this is in agreement with the study of McClung et al. [41].

In the present study, hepatic TBARS level was increased in the HFD group. It is reported that hyperglycemia and hypertriglyceridemia evoke the hepatic lipid peroxidation due to *β*-oxidation overload that is characterized by high TBARS level [42]. Also level of GSH, GST, and NO are elevated in HFD group.

*Berberis vulgaris* L. is considered as one of the well-known medicinal plants with traditional herbal medical history and is used in many civilizations as a curative herbal remedy in the homeopathic system of medicine [12]. The most important constituents are isoquinoline alkaloids, such as berberine, berbamine, and palmatine [43]. Berberine represents one of the most studied naturally occurring protoberberine alkaloids, since it possesses a wide range of biochemical and pharmacological activities [44, 45].

Berberis crude extract showed a protective and/or curative capacity with markedly hypoglycemic, hypolipidemic, and antioxidant properties against IR incidence and progression.

Our data showed that oral administration of berberine reduced the key features of insulin resistance syndrome. This is in agreement with several studies that have demonstrated the role of berberine in the protection against HFD induced insulin resistance [14, 46, 47]. This is illustrated when using berberine as a treatment for 2 weeks normalized insulin, glucose, and HOMA-IR levels in both treated and protected groups when compared to HFD group. The observed hypoglycemic effects of berberine are supported by the previous in vitro [48, 49] and in vivo studies [50, 51]. Also, Gomes et al., Kalalian-Moghaddam et al., and El-Sayed et al. [52–54] revealed that berberine supplementation has a positive impact on glucose and insulin homeostasis upon HFD feeding. Also, levels of cholesterol, triglyceride, and LDL-cholesterol are reduced in addition to increased HDL-cholesterol level. These results are supported by other studies that showed the same effect of berberine on lipid profile [53, 54].

The phytochemical constituents of berberine as alkaloids, flavonoids, and phenolic contents would act in synergy in order to increase barberry's bioactivity such as antioxidant and antidiabetic. So, the protection or the treatment with berberine maintained the antioxidant enzymes in the normal level. This is in agreement with the study of El-Sayed et al. [52].

Also, BRB has an effect on proinflammatory cytokines, as after treatment with berberine there is a stimulatory effect on IL-12 and an inhibitory effect on IL-6 production compared to untreated HFD group. From our data we found that berberine has an effect on IFN-*γ* production, as after treatment for 2 weeks the level of IFN-*γ* was improved compared to HFD group. These findings come in agreement with the study of Aziz et al. [55] which said that BRB has immune modulatory effect on immune system and after treatment of DCs, there is an increase in level of INF-*γ* and IL-12.

The present study showed that there is a relation between IR and production of IFN-*γ*, also showing the powerful effect of berberine as an inhibitor for insulin resistance syndrome development and its usage as a protective compound indicating its importance for people who are infected with viral infection like virus C.

## Figures and Tables

**Table 1:** Composition of HFD.

Ingredients	Diet (g/kg)
Powdered NPD	365
Butter	310
Casein	253
Cholesterol	10
Vitamin and mineral mix	60
Yeast powder	1
Sodium chloride	1

**Table 2:** Effect of HFD alone or combined with berberine on insulin resistance.

Groups	Body weight (gm)	Glucose (mg/dL)	Insulin (*µ*U/mL)	HOMA-IR	HOMA-*β*
Control	149.16 ± 10.88^a^	95.70 ± 9.70^a^	11.0 ± 1.43^a^	2.209 ± 0.52^a^	153 ± 11.6^a^
Untreated HFD	178.5 ± 14.33^b^	130.45 ± 15.29^b^	21.8 ± 2.77^b^	6.09 ± 1.33^b^	102.86 ± 23.2^b^
Treated HFD with BRB	157.66 ± 11.80^a^	107.08 ± 3.80^a^	11.7 ± 1.16^a^	2.73 ± 0.61^a^	118 ± 8.6^a^
Protected HFD with BRB	173.5 ± 15.87^b^	110.95 ± 4.27^a^	11.9 ± 1.78^a^	2.72 ± 0.91^a^	93.4 ± 5.7^a^

Mean value in each column having different superscript (a, b) is significant difference at *p* < 0.05.

**Table 3:** Effect of HFD alone or combined with berberine on lipid profile.

Groups	Cholesterol (mg/dL)	Triglyceride (mg/dL)	HDL-chol (mg/dL)	LDL-chol (mg/dL)
Control	100.06 ± 12.60^a^	68.72 ± 14.61^a^	66.20 ± 8.93^a^	28.22 ± 4.56^a^
Untreated HFD	202.05 ± 21.96^b^	165.72 ± 7.11^b^	49.30 ± 6.7^b^	119.61 ± 14.61^b^
Treated HFD with BRB	164.66 ± 15.56^c^	123.47 ± 11.04^c^	55.5 ± 6.88^c^	84.46 ± 8.17^c^
Protected HFD with BRB	180.68 ± 15.84^c^	145.09 ± 14.70^c^	55.0 ± 5.27^c^	96.66 ± 10.32^c^

Mean value in each column having different superscript (a, b, and c) is significant difference at *p* < 0.05.

**Table 4:** Effect of HFD alone or combined with berberine on cytokines profile, WBCs, and lymphocytes count.

Groups	IL-6 (pg/mL)	IL-12 (ng/mL)	IFN-*γ* (pg/mL)	WBC *∗* 10^3^	LYMF
Control	80.21 ± 4.13^a^	159.96 ± 13.16^a^	91.72 ± 11.85^a^	6.7 ± 1.51^a^	3.64 ± 0.47^a^
Untreated HFD	143.75 ± 13.62^b^	105.58 ± 13.92^b^	21.16 ± 1.11^b^	5.6 ± 1.71^a^	1.75 ± 0.71^b^
Treated HFD with BRB	91.08 ± 3.61^c^	153.46 ± 16.96^a^	49.32 ± 5.55^c^	5.16 ± 1.11^a^	2.15 ± 0.9^c^
Protected HFD with BRB	117.01 ± 7.92^d^	148.19 ± 18.79^a^	46.90 ± 6.17^c^	5.96 ± 1.85^a^	2.0 ± 0.66^c^

Mean value in each column having different superscript (a, b, c, and d) is significant difference at *p* < 0.05.

**Table 5:** Effect of HFD alone or combined with berberine on liver oxidative stress parameters level.

Groups	TBARS (nmol/mL)	GSH (mg/mL)	GST (*µ*mol/min)	NO (*µ*mol)
Control	4.25 ± 0.159^a^	0.187 ± 0.01^a^	0.121 ± 0.01^a^	72.39 ± 36.26^a^
Untreated HFD	5.25 ± 0.113^b^	0.208 ± 0.01^b^	0.148 ± 0.01^b^	163.66 ± 23.42^b^
Treated HFD with BRB	4.28 ± 0.166^a^	0.188 ± 0.01^a^	0.126 ± 0.01^a^	92.39 ± 70.77^a^
Protected HFD with BRB	4.42 ± 0.161^a^	0.191 ± 0.01^a^	0.136 ± 0.01^a^	95.78 ± 54.84^a^

Mean value in each column having different superscript (a, b) is significant difference at *p* < 0.05.
